# A Peak Current Mode Boost DC-DC Converter with Hybrid Spread Spectrum

**DOI:** 10.3390/mi16080862

**Published:** 2025-07-26

**Authors:** Xing Zhong, Jianhai Yu, Yongkang Shen, Jinghu Li

**Affiliations:** 1 School of Computer, Electronics and Information, Guangxi University, Nanning 530004, China; 2213391016@st.gxu.edu.cn (X.Z.); 2313393046@st.gxu.edu.cn (Y.S.); 2 The Guangxi Key Laboratory of Machine Vision and Intelligent Control, Wuzhou University, Wuzhou 543002, China; 3 The College of Computer and Information Sciences, Fujian Agricultural and Forestry University, Fuzhou 350002, China; lily_hit@126.com

**Keywords:** boost converter, pulse width modulation, spread spectrum, integrated circuit

## Abstract

The stable operation of micromachine systems relies on reliable power management, where DC-DC converters provide energy with high efficiency to extend operational endurance. However, these converters also constitute significant electromagnetic interference (EMI) sources that may interfere with the normal functioning of micro-electromechanical systems. This paper proposes a boost converter utilizing Pulse Width Modulation (PWM) with peak current mode control to address the EMI issues inherent in the switching operation of DC-DC converters. The converter incorporates a Hybrid Spread Spectrum (HSS) technique to effectively mitigate EMI noise. The HSS combines a 1.2 MHz pseudo-random spread spectrum with a 9.4 kHz triangular periodic spread spectrum. At a standard switching frequency of 2 MHz, the spread spectrum range is set to ±7.8%. Simulations conducted using a 0.5 μm Bipolar Complementary Metal-Oxide-Semiconductor Double-diffused Metal-Oxide-Semiconductor (BCD) process demonstrate that the HSS technique reduces EMI around the switching frequency by 12.29 dBμV, while the converter’s efficiency decreases by less than 1%.

## 1. Introduction

Owing to their high energy conversion efficiency and strong load capacity, DC-DC converters are extensively utilized in various applications, including automotive electronics, portable electronic devices, communication systems, and micromachine systems [1,2,3,4,5,6,7,8]. For instance, a wireless sensor network typically consists of DC-DC converters, sensing circuits, clock modules, and radio frequency modules [9]. Since all of these modules are integrated into a smaller printed circuit board to reduce size and increase power density, the EMI generated by the normal operation of the DC-DC converter may affect the performance of other noise-sensitive circuits, such as radio frequency modules. In addition, accelerometers, gyroscopes, microphones, and other micromachine devices are susceptible to EMI noise generated by switching DC-DC converters and radio frequency circuits, which can degrade detection accuracy or cause signal distortion. Consequently, it is crucial to implement strategies to attenuate the EMI produced by DC-DC converters.

In recent decades, researchers have investigated numerous strategies to address the electromagnetic interference (EMI) challenges posed by DC-DC converters, one of which is inserting EMI filters into the input port of DC-DC converters. While passive LC filtering demonstrates effective EMI suppression, its requirement for μF-level capacitors and μH-level inductors presents significant implementation challenges: these bulky components cannot be integrated on-chip, necessitating external passives. Thus, this method increases cost and area, which is not conducive to improving power density, and affects the system response of the converter [10,11,12,13]; another technique focuses on reducing the slew rate by adding resistors to the gate of switching power transistors. Although this method lowers EMI while enabling full on-chip integration, it comes at the expense of higher switching losses, which reduces the converter’s energy conversion efficiency to some extent [14,15,16]. Alternative approaches include package modification, PCB layout optimization, zero-voltage switching (ZVS), multilevel converter topologies, and variable switching frequency control techniques [17,18,19,20,21,22,23,24,25].

**Figure 1:**
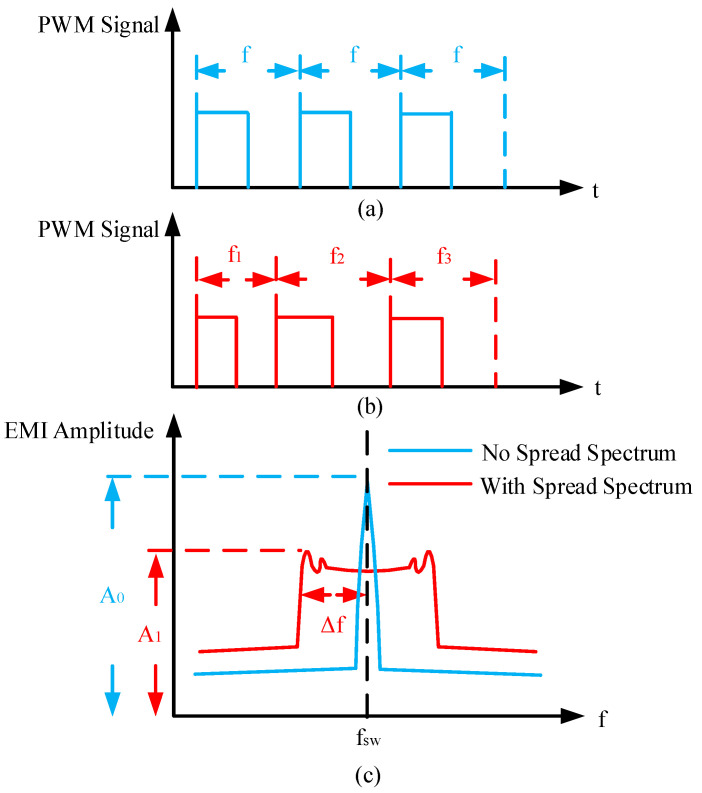
(**a**) PWM-controlled; (**b**) PWM-controlled with spread spectrum; (**c**) EMI reduction with and without spread spectrum.

Diverging from the previously discussed approaches, for PWM-controlled DC-DC converters, several papers have demonstrated that clock spread spectrum technology, derived from digital communication, can reduce the EMI of PWM DC-DC converters [26,27] and that spread spectrum technology can effectively reduce EMI without the need for off-chip components, making it a cost-effective solution. In addition, the spread spectrum is centered on the original switching frequency, and the frequency expansion is ±16% in a small range, which has little impact on the voltage regulation performance and energy conversion efficiency of converters. According to the Carson rule described in [28], the total energy of EMI remains constant before and after applying spread spectrum. However, the EMI is redistributed over a wider frequency range, which reduces the peak amplitude of EMI in frequency bands, as shown in Figure 1. The effectiveness of spread spectrum techniques in EMI suppression is closely related to the modulation pattern employed. Currently, the spread spectrum profiles used for DC-DC converters primarily include sinusoidal, triangular, exponential, chaotic, and random modulation [29,30,31,32].

Current research predominantly focuses on applying spread spectrum techniques to buck converters, with limited investigation into boost converter implementations. In this paper, we propose an HSS technique and implement it in a peak-current-mode boost converter, which combines the characteristics of the simplest implementation method of the triangular spread spectrum and the flattest frequency spectrum of a random pattern, and superimposes a pseudo-random spread spectrum of 1.2 MHz on the 9.4 kHz triangular spread spectrum. In addition, this paper also implements the HSS using analog control loops and digital methods, combining the advantages of the analog control loops, such as simple structure and fast response speed, with those of digital spread spectrum, including good controllability and being less affected by external conditions. This article provides a feasible on-chip solution for EMI reduction of boost converters for portable applications and micromachine systems. The structure of this paper is as follows: Section 2 presents the spread spectrum explanation and HSS design; Section 3 shows the simulation results; Section 4 offers a discussion; and finally, conclusions are given in Section 5.

## 2. Materials and Methods

### 2.1. Spread Spectrum Explanation

For typical PWM-controlled boost converters, the switching frequency is fixed at a specific frequency, as shown in Figure 1a. This results in EMI noise being predominantly concentrated within a narrow frequency band, leading to high amplitude peaks. The spread spectrum expands the original single switching frequency to several frequencies, and the EMI noise is redistributed to a wider frequency band, according to the Carson rule, so the amplitude of the EMI noise after spread spectrum is reduced, as shown in Figure 1c. An important parameter describing the spread spectrum characteristics is the modulation factor *m*, which is defined as follows:

(1) $$m=\frac{\Delta f_{sw}}{f_{m}}$$

Among them, Δ*f_sw_* represents the spread frequency range of the switching frequency, *f_m_* denotes the modulation frequency, and the relationship between EMI noise reduction and m is as follows [9]:

(2) ΔEMI≈logm

To achieve significant EMI reduction, it is necessary to increase m. This can be accomplished by either increasing Δ*f_sw_* or decreasing *f_m_*. However, practical limitations exist for both approaches. Increasing Δ*f_sw_* can negatively impact the voltage regulation performance of the converter, which is why Δ*f_sw_* is typically limited to ±16%. On the other hand, decreasing *f_m_* below the bandwidth of the EMI measuring instrument can interfere with accurate EMI measurement. Therefore, it is crucial to select m judiciously to strike a balance between maintaining voltage regulation performance and achieving effective EMI noise reduction. In this design, the spread spectrum range is set to ±7.8%, the modulation frequency is 9.4 kHz, resulting in a modulation factor of m = 16.6. This configuration achieves an EMI reduction of approximately 12.38 dBμV.

### 2.2. Spread Spectrum Design

**Figure 2:**
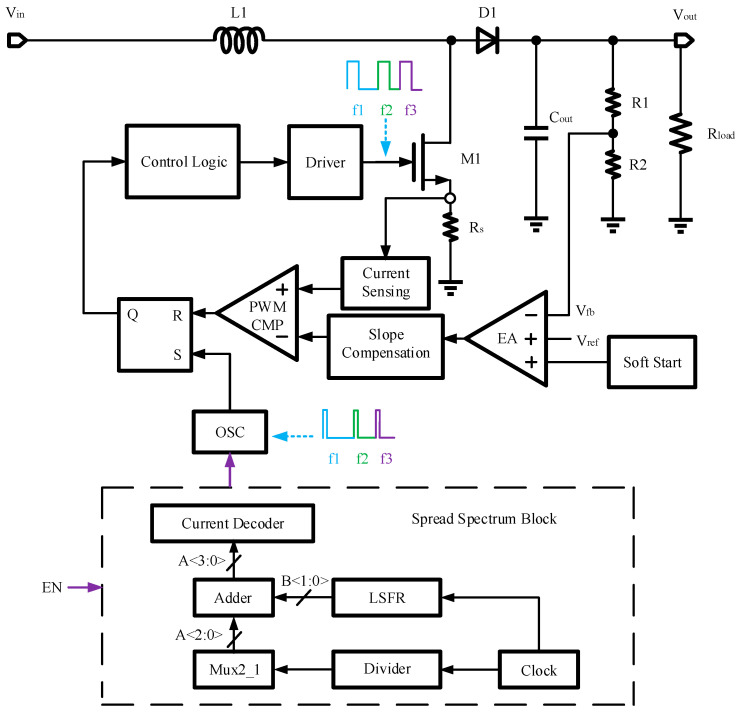
The overall structure of the boost converter with HSS in this paper.

The boost converter with PWM control and peak current mode, along with the spread spectrum module devised in this paper, is depicted in Figure 2. This system is primarily structured around a power stage, a control loop, and a spread spectrum module. The control loop encompasses an error amplifier (EA), an inductor current sensing circuit (CS), a slope compensation circuit (SC), a PWM comparator (PWM CMP), an RS latch, an oscillator (OSC), a control logic circuit, and a driver. Auxiliary modules include a bandgap reference (BGR), a low dropout regulator (LDO), and a soft start circuit (SS). The spread spectrum module is chiefly made up of a clock, a binary number generator, and a current decoder, which collectively modulate the switching frequency of the converter by adjusting the oscillator’s frequency. This research employs the HSS technique, setting the spread spectrum depth at ±7.8% of the original switching frequency with a step size of 1.5%, totaling 11 steps. Moreover, the design adopts the digital spread spectrum, offering superior controllability, reduced power consumption, and greater resilience to external environmental influences compared to analog methods.

### 2.3. Oscillator

**Figure 3:**
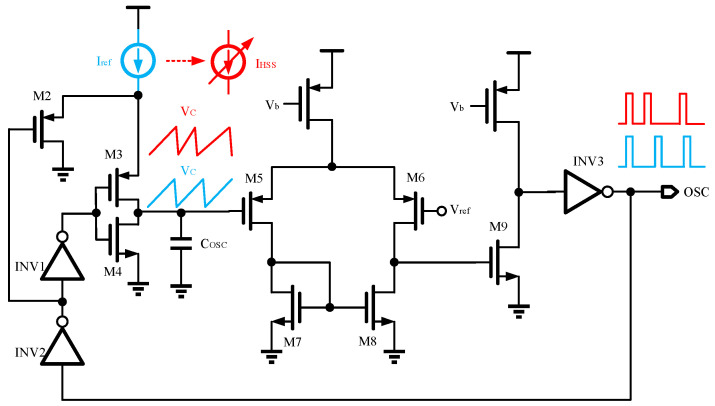
The oscillator schematic used in this article.

In PWM-controlled and peak current mode converters, an oscillator is typically integrated to define the PWM period and turn on switch transistors [33,34]. The oscillator circuit utilized in this study is depicted in Figure 3. It comprises a comparator, a capacitor, current sources, switches, and inverters. Here, *V_ref_* and *I_ref_* denote the reference voltage and current derived from BGR. The comparator is constructed using an open-loop two-stage operational amplifier, with its negative and positive inputs connected to *V_ref_* and the capacitor, respectively. The operational principle of the oscillator involves managing the charging and discharging of the capacitor via the comparator to generate a narrow pulse at a fixed frequency [35,36]. The details are as follows: Initially, presume the capacitor *C_osc_* holds no charge. *I_ref_* begins to charge *C_osc_*, causing the voltage across it, which denotes *V_C,osc_*, to increase linearly. As long as this voltage does not exceed *V_ref_*, the comparator’s output remains high, which, after inversion, keeps the transistor *M4* off. This charging phase lasts for a duration *t1*. Secondly, when *V_C,osc_* exceeds *V_ref_*, the comparator output flips to low, the comparator’s output flips to low, which inverts to high, turning on *M4*. *M4* then operates in the linear region, presenting a small equivalent resistance *R*, significantly less than that of the charging current source. This results in a rapid discharge of *C_osc_*, reducing the voltage across it to zero in a time *t2*. After these two phases, the oscillator generates a narrow pulse waveform. The total charge and discharge period of the oscillator is *t1* + *t2*. Since the discharge current is much larger than the charging current, *t1* is significantly greater than *t2*. If *t2* is negligible, based on the voltage and current integration relationship of *C_osc_*, as shown in Equation (3), the oscillation frequency *f_osc_* of the oscillator can be approximated as:

(3) $$V_{ref}=\int_{0}^{t1}\frac{I_{ref}}{C_{osc}}dt$$

(4) $$f_{osc}=\frac{1}{t1}=\frac{I_{ref}}{C_{osc}V_{ref}}$$

From Equation (4), it is clear that the oscillation frequency of the oscillator, which determines the switching frequency of the PWM converter, depends on *I_ref_*, *V_ref_*, and *C_osc_*. By adjusting any one of these parameters, the switching frequency can be modulated, enabling spread-spectrum functionality. In this study, the spread spectrum is achieved by dynamically varying *I_ref_*, as the spread spectrum frequency is directly proportional to *I_ref_*. The clock, binary number generator, and current decoder are used to change the reference current to achieve HSS.

### 2.4. Clock

**Figure 4:**
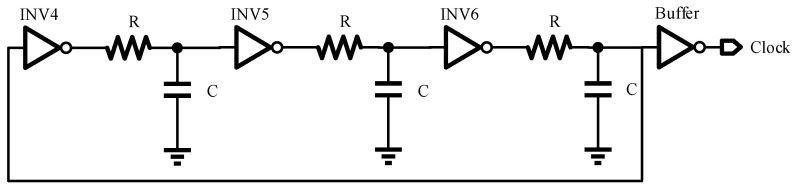
Clock circuit.

Since this article employs the digital spread spectrum approach, a clock is essential to ensure the proper operation of digital circuits such as D flip-flops. The clock, composed of a cascade of 3-stage inverters with a simple structure and low power consumption, is used. However, the frequency of a clock based solely on inverters is typically too high for the desired application. In this work, the target pseudo-random spread frequency is set to 1.2 MHz. Therefore, *RC* circuits are incorporated into each stage of the inverter to reduce the clock frequency. The circuit diagram is illustrated in Figure 4. The flip threshold of the inverter is set to half of the supply voltage, because the delay of the *RC* circuit is much greater than the transmission delay of the inverter, so the calculation formula of the clock frequency can be simplified as follows:

(5) $$f_{clock}=\frac{1}{6T_{D,RC}}=\frac{1}{6RC\ln2}$$

### 2.5. Binary Number Generator

**Figure 5:**
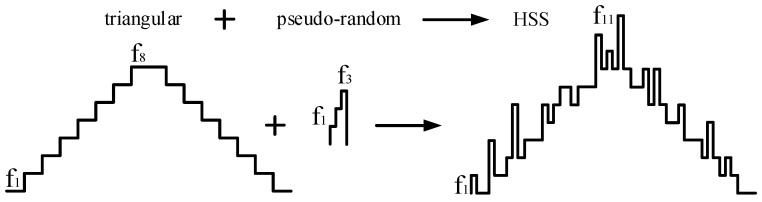
HSS allocation.

**Figure 6:**
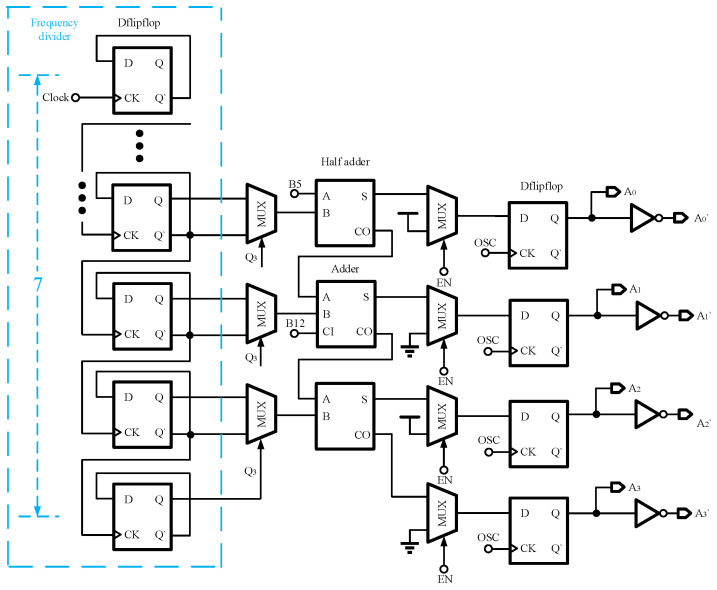
4-bit binary numbers generator implementation.

**Figure 7:**
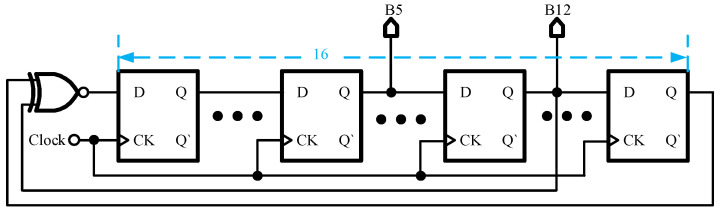
16-bit LFSRs.

In this design, the digital HSS is configured with a spread spectrum range of ±7.8%, a step size of 1.5%, and a total of 11 steps. To achieve these 11 steps, a binary encoding method requiring at least 4 binary digits is employed. Specifically, the periodic triangular spread spectrum is set to 8 steps, while the pseudo-random spread spectrum is set to 4 steps, with one repeating state removed, resulting in a total of 11 steps. The HSS allocation is shown in Figure 5. This is accomplished using 3-bit binary numbers for the periodic triangular spread spectrum and 2-bit binary numbers for the pseudo-random spread spectrum. These are then merged to form 4-bit binary numbers. The circuit diagram of the whole binary number generator is shown in Figure 6. First, for the periodic triangular spread spectrum, this design employs a 128-1 frequency divider composed of 7 D flip-flops to divide the clock signal, generating 3-bit binary numbers. As the divider counts from 000 to 111, three 2-to-1 data selectors are used to reverse the counting direction, causing the divider to transition from 111 back to 000. This process repeats, creating 3-bit binary numbers with a periodic triangular envelope of 9.4 kHz. In the context of pseudo-random spread spectrum, a commonly employed method utilizes the Linear Feedback Shift Registers (LFSRs) to generate the desired signal. As illustrated in Figure 7, 16-bit LFSRs are implemented in this study to ensure sufficient randomness. Specifically, the 5th and 12th bits of the LFSR are extracted and combined using adders to produce a 3-bit binary sum. This process yields 4-bit random binary numbers characterized by a periodic triangular envelope and pseudo-random properties. The outputs vary within the range of 0000 to 1011, encompassing 11 discrete steps, thereby achieving an initial implementation of HSS. Moreover, the original switching frequency is encoded as 0101; that is, when the HSS is not used, the pseudo-random generation module outputs 0101. In addition, in order to avoid binary numbers changing in one cycle of the oscillator, four D flip-flops are required for synchronization.

### 2.6. Current Decoder

**Figure 8:**
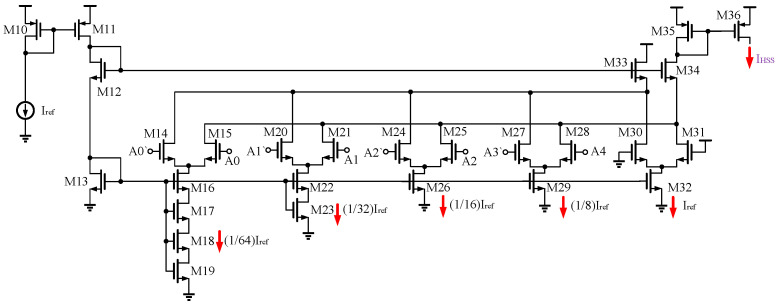
The schematic of the current decoder.

The current decoder serves the purpose of translating *I_ref_* into the corresponding HSS current based on binary numbers, which involves the conversion of digital signals to analog signals, so a digital to analog converter (DAC) is required, and among various DACs, the current steering DAC is the most suitable for this design because of its fast conversion speed and natural output of analog current signals [37]. To accommodate the 1.5% spread spectrum step utilized in this design, a 6-bit current steering DAC with a resolution of 1/64 is employed. However, this DAC requires modification to align with the ±7.8% spread spectrum range proposed in this paper. To achieve this, the 5th and 6th bits (corresponding to 1/2 and 1/4 of *I_ref_*) are removed, and a 5th bit set to 1 (denoting *I_ref_*) is introduced. The circuit diagram of the modified current decoder is illustrated in Figure 8. The output current of the current decoder is as follows:

(6) $$I_{HSS}=I_{ref}+A_{3}8I_{u}+A_{2}4I_{u}+A_{1}2I_{u}+A_{0}I_{u}$$

Here, *I_u_* = 1/64*I_ref_*. The maximum and minimum values of I_HSS_ are *I_ref_* + 15.6% and *I_ref_*, respectively, resulting in an adjustment range of 15.6% with a step size of 1.5%. The switching frequency without spread spectrum is assigned the corresponding random number 10101, which corresponds to *I_ref_* + 7.8%. From Equation (4), modulating the oscillator reference current positively correlates the switching frequency, so that a spread spectrum of ±7.8% of the original switching frequency is achieved in the HSS profile, which is determined by 5-bit binary numbers, as follows:

(7) $$f_{sw}=\frac{I_{HSS}}{C_{osc}V_{ref}}=\frac{I_{ref}+A_{3}8I_{u}+A_{2}4I_{u}+A_{1}2I_{u}+A_{0}I_{u}}{C_{osc}V_{ref}}$$

## 3. Simulation Results

**Figure 9:**
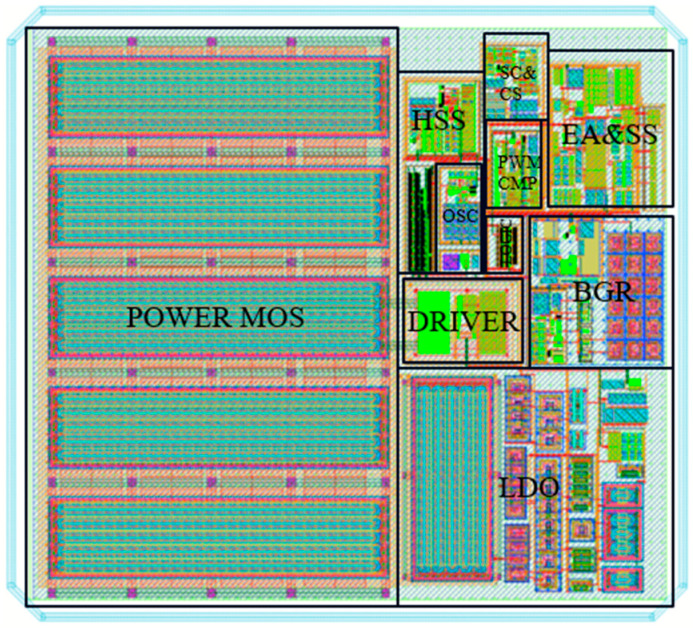
The layout of the boost converter with HSS proposed in this paper.

The peak current mode boost DC-DC converter introduced in this paper is designed using the 0.5 μm BCD process. It accommodates an input voltage range of 4 V to 9 V and delivers a fixed output voltage of 12 V, with a load current capability of 0 to 1 A. The converter operates exclusively in PWM mode, with a central switching frequency of 2 MHz and a spread spectrum range of ±7.8%. The power inductor and output capacitor are 4.7 μH and 12 μF. The key parameters of the boost converter are summarized in Table 1. The layout of the converter is depicted in Figure 9, where the active area of the design is 997 μm × 1106 μm.

**Figure 10:**
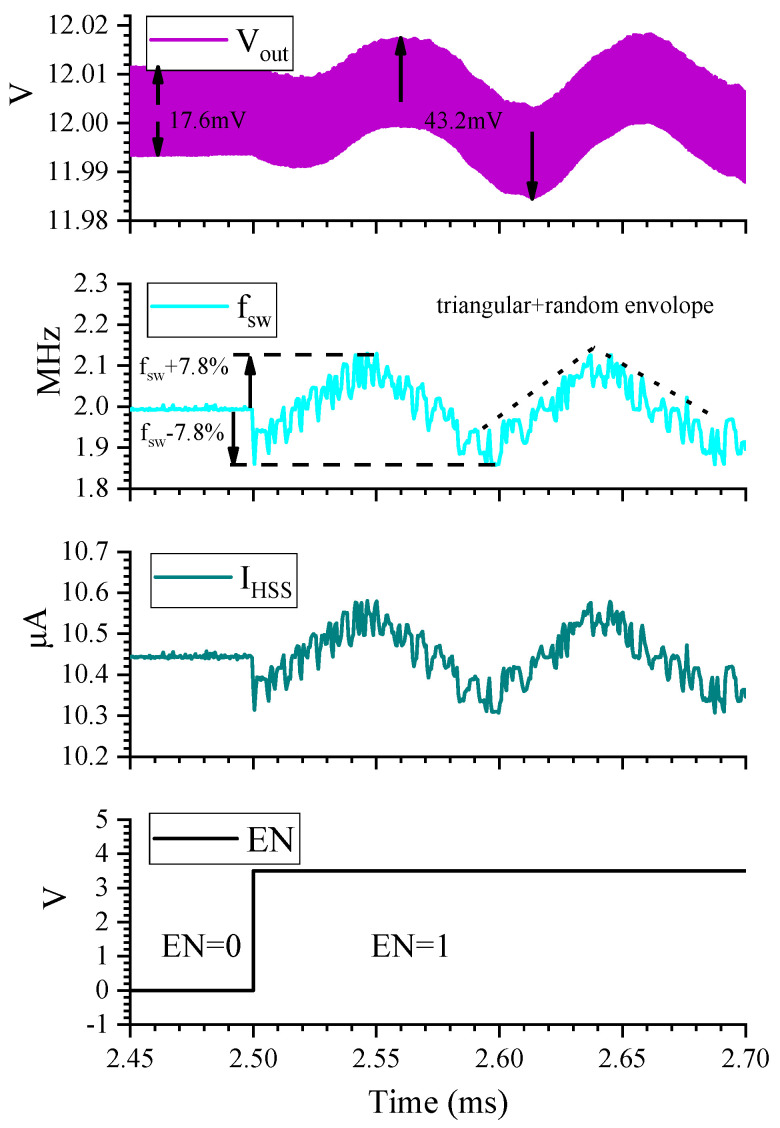
Key waveforms for enabling and disabling HSS.

Figure 10 illustrates the steady-state waveforms of the output voltage *V_out_*, *I_HSS_*, and *f_sw_* of the proposed boost converter over time t. The simulations are taken under the conditions of a 6 V input and a 12 V output. The HSS enable signal (EN) transitions from logic 0 to 1 at 2.5 ms, activating the HSS. Once HSS is enabled, binary numbers change in a triangular and pseudo-random envelope, causing *I_HSS_* to exhibit a positive correlation change. Consequently, *f_sw_* also follows this pattern. Notably, the spread spectrum introduces jitter on the output voltage, and the jitter pattern is correlated with the spread spectrum mode. This jitter slightly degrades the voltage regulation performance of the converter [38].

**Figure 11:**
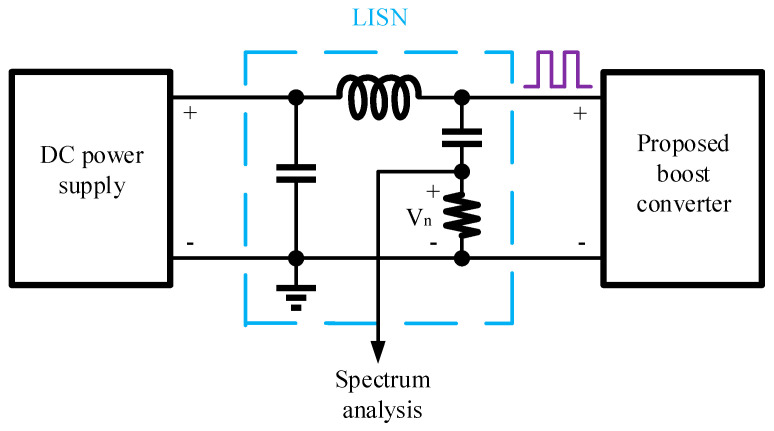
Conducted an EMI simulation method.

**Figure 12:**
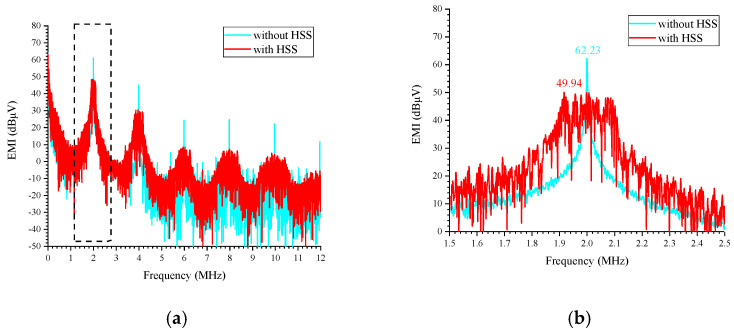
Simulated Conducted EMI reduction with and without spread spectrum: (**a**) from 0 to 12 MHz; (**b**) zoom in view from 1.5 to 2.5 MHz.

Based on the conducted EMI simulation method described in [14], a Line Impedance Stabilization Network (LISN) is inserted between the power supply and the converter, as depicted in Figure 11. The LISN is used to simulate the noise voltage generated across its 50 Ω resistor. By performing a spectrum analysis of this noise voltage, the conducted EMI can be evaluated. Figure 12 presents the conducted EMI results for the boost converter, both with and without HSS enabled, under the conditions of a 6 V input, a 12 V output, a 0.5 A load current, and a switching frequency of 2 MHz. The results demonstrate that the implementation of HSS reduces the conducted EMI by 12.29 dBμV.

**Figure 13:**
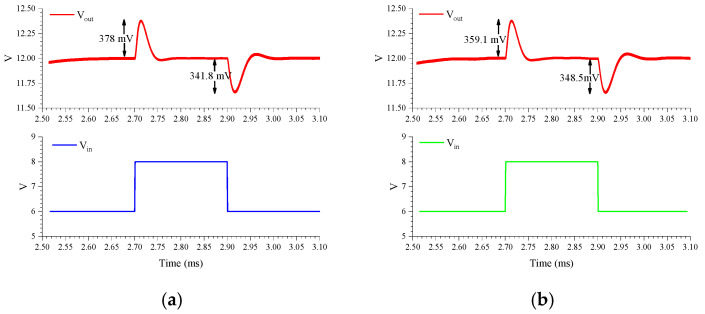
Linear transient response with: (**a**) PWM-controlled boost converter; (**b**) PWM-controlled boost converter with HSS.

**Figure 14:**
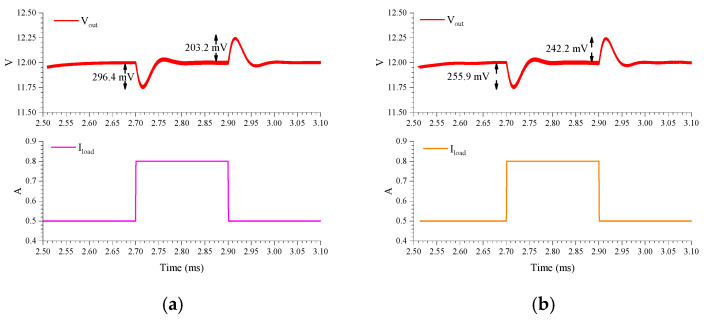
Load transient response with: (**a**) PWM-controlled boost converter; (**b**) PWM-controlled boost converter with HSS.

In order to verify that the HSS exerts minimal influence on the dynamic response characteristics of the boost converter, simulations of the converter’s linear transient response have been conducted. Figure 13a shows the transient response of the PWM-controlled boost converter with *V_in_* switching between 6 V and 8 V in 1μs. The overshoots and undershoots are 378 mV and 341.8 mV, respectively. In Figure 13b, the overshoot and undershoot of the PWM-controlled boost converter with HSS are 359.1 mV and 348.5 mV. Similarly, Figure 14a,b present the load transient responses of the standard PWM-controlled boost converter and the HSS-incorporated boost converter, respectively. The simulation results demonstrate that the overshoot, undershoot, and settling behavior of the boost converter with HSS exhibit few differences compared to those without HSS. Therefore, the HSS does not significantly affect the transient response performance of the boost converter.

**Figure 15:**
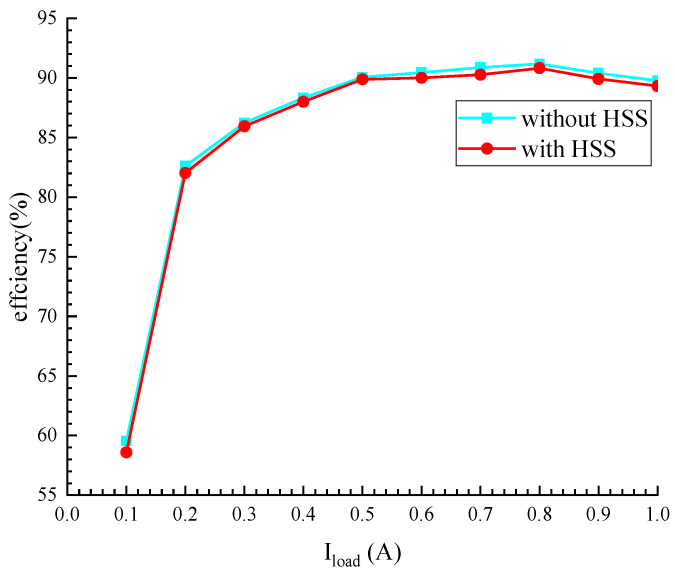
Simulated efficiency with and without spread spectrum.

Figure 15 provides a comparison of the conversion efficiency of the converter with HSS enabled and non-HSS enabled in the range of 0–1 A load current, the input voltage is 6 V, and the output voltage is 12 V, because this paper uses a digital spread spectrum module, which consume relatively low power, and it can be seen that the efficiency after using spread spectrum is reduced by less than 1%. In addition, Table 2 compares the performance of this paper and other references. Compared to conventional EMI suppression approaches, such as EMI filters and slew rate control, the proposed HSS technique effectively addresses their inherent limitations. Notably, the HSS implementation does not require off-chip components while maintaining power efficiency. In terms of EMI mitigation effectiveness, the proposed HSS demonstrates comparable performance in boost converters to what spread spectrum achieves in buck converters, thereby validating its efficacy for boost converter applications. This work, therefore, presents a viable on-chip solution for EMI reduction in boost converters.

## 4. Discussion

Compared to conventional EMI suppression approaches involving filtering and slew rate reduction, spread spectrum techniques offer distinct advantages: (1) elimination of off-chip passive components, (2) minimal efficiency degradation (<2%), and (3) compatibility with power density optimization and cost reduction. These characteristics establish spread spectrum as an effective solution for EMI mitigation in DC-DC converters. However, the spread spectrum technology also has its shortcomings, as mentioned in Section 3, the spread spectrum has a little impact on the voltage regulation performance of the converter. Both references [5] and [35] exhibit similar output voltage jitter phenomena. In general, DC-DC converters can tolerate output voltage jitter within 1% of the nominal output. Therefore, the spread spectrum range is typically limited to ±16% of the nominal switching frequency. This constrained frequency deviation ensures minimal impact on output voltage regulation while still providing effective EMI suppression. The root cause of output voltage jitter lies in the fact that HSS modulation of the switching frequency simultaneously affects the duty cycle (*D*). The relationship between *D* and switching frequency is given by:

(8) $$D=\frac{t_{on}}{T_{sw}}=t_{on}f_{sw}$$

where *t_on_* represents the switch conduction time per cycle. This demonstrates that switching frequency jitter directly induces correlated duty cycle variations. While the implemented peak current mode control establishes a negative feedback loop to attenuate duty cycle jitter, complete jitter elimination remains unachievable. In PWM boost converters, the output voltage exhibits dependence on *D* as follows:

(9) $$V_{out}=\frac{V_{in}}{1-D}$$

Consequently, HSS generates output voltage jitter with a triangular envelope characteristic. So, some methods need to be taken to suppress the jitter caused by the duty cycle.

## 5. Conclusions

In this paper, we propose a peak current mode boost converter integrated with HSS to reduce EMI. The HSS combines periodic triangular and pseudo-random spread spectrum, effectively lowering EMI noise. Simulation results demonstrate that the HSS reduces EMI by 12.29 dBμV while maintaining an efficiency loss of less than 1% compared to the non-spread spectrum case.

## Figures and Tables

**Table 1:** Specification of the proposed boost converter.

Parameter	Value
Input voltage	4 V–9 V
Output voltage	12 V
power inductor	4.7 μH
output capacitor	12 μF
load current	0–1 A
switching frequency	2 MHz
spread spectrum range	±7.8%

**Table 2:** Performance comparison.

	[39]	[9]	[40]	[41]	This Work
Year	2016	2022	2023	2024	2025
Topology	Buck	Buck	Buck	Buck	Boost
EMI suppression method	Slew rate control	Spread spectrum	Spread spectrum	EMI filter	Spread spectrum
Need of-chip components	No	No	No	Yes	No
Process (μm)	/	0.18	0.18	/	0.5
Switching frequency (MHz)	/	2	1.5	/	2
spread spectrum way	/	Onion	Triangular	/	HSS
spread spectrum range (%)	/	±10	±5	/	±7.8
EMI attenuation (dBμV)	20	12	11.5	30	12.29
Efficiency loss (%)	<3	/	<1	/	<1
Output voltage jitter (mV)	/	43	40	/	43.2

## Data Availability

The data presented in this study are available on request from the corresponding author.

## References

[1] Park Y.-J., Park J.-H., Kim H.-J., Hocheol R., Kim S.Y., Pu Y.G. (2017). A Design of a 92.4% Efficiency Triple Mode Control DC–DC Buck Converter with Low Power Retention Mode and Adaptive Zero Current Detector for IoT/Wearable Applications. IEEE Trans. Power Electron..

[2] Tekin H., Setrekli G., Murtulu E., Karşıyaka H., Ertekin D. (2023). A Proposed Single-Input Multi-Output Battery-Connected DC–DC Buck–Boost Converter for Automotive Applications. Electron.

[3] Touré M.L., Camara M.B., Dakyo B. (2024). Symmetrical Multilevel High Voltage-Gain Boost Converter Control Strategy for Photovoltaic Systems Applications. Electronics.

[4] Monteiro J., Pires V.F., Foito D., Cordeiro A., Silva J.F., Pinto S. (2023). A Buck-Boost Converter with Extended Duty-Cycle Range in the Buck Voltage Region for Renewable Energy Sources. Electronics.

[5] Kok C.L., Siek L. (2024). Designing a Twin Frequency Control DC-DC Buck Converter Using Accurate Load Current Sensing Technique. Electronics.

[6] Kok C.L., Tang H., Teo T.H., Koh Y.Y. (2024). A DC-DC Converter with Switched-Capacitor Delay Deadtime Controller and Enhanced Unbalanced-Input Pair Zero-Current Detector to Boost Power Efficiency. Electronics.

[7] Tan C.B., Siek L. (2024). A 10 V-to-1 V Double Step-Down Buck Converter Using Time-Based Current Mode Control with Minimum Delay Frequency Difference Phase Adder for 1 MHz Operation. J. Low Power Electron. Appl..

[8] Wu C., Zhang J., Zhang Y., Zeng Y. (2023). A 7.5-mV Input and 88%-Efficiency Single-Inductor Boost Converter with Self-Startup and MPPT for Thermoelectric Energy Harvesting. Micromachines.

[9] Huang H.-Y., Villaruz H.M., Mapula N.M. (2022). High-Efficiency Low-EMI Buck Converter Using Multistep PWL and PVT Insensitive Oscillator. IEEE Trans. Power Electron..

[10] Chu Y., Wang S., Wang Q. (2016). Modeling and Stability Analysis of Active/Hybrid Common-Mode EMI Filters for DC/DC Power Converters. IEEE Trans. Power Electron..

[11] Zhang Y., Jiang D. (2022). An Active EMI Filter in Grounding Circuit for DC Side CM EMI Suppression in Motor Drive System. IEEE Trans. Power Electron..

[12] Zhai L., Hu G., Lv M., Zhang T., Hou R. (2020). Comparison of Two Design Methods of EMI Filter for High Voltage Power Supply in DC-DC Converter of Electric Vehicle. IEEE Access.

[13] Bai Y., Hu S., Tahir M., Yang Z., Zhi Y. (2024). An Active EMI Filter Using Predictive Pulsed Compensation for Common Mode Noise Suppression of Boost Converters. IEEE J. Emerg. Sel. Top. Power Electron..

[14] Ke X., Sankman J., Chen Y., He L., Ma D.B. (2018). A Tri-Slope Gate Driving GaN DC–DC Converter with Spurious Noise Compression and Ringing Suppression for Automotive Applications. IEEE J. Solid-State Circuits.

[15] Ding Y., Zhu C., Gu J., Zhang Z., Lu Y., Xu Y. (2024). A Cost-Efficient Active Gate Driver for Seamless Slew Rate Control of SiC MOSFETs. IEEE Trans. Power Electron..

[16] Yang Y., Huang M., Du S., Martins R.P., Lu Y. (2023). A Level Shifter with Almost Full Immunity to Positive dv/dt for Buck Converters. IEEE Trans. Circuits Syst. I Regul. Pap..

[17] Mukherjee R., Patra A., Banerjee S. (2010). Impact of a Frequency Modulated Pulsewidth Modulation (PWM) Switching Converter on the Input Power System Quality. IEEE Trans. Power Electron..

[18] Ma Z., Wang S., Sheng H., Lakshmikanthan S. (2023). Modeling, Analysis and Mitigation of Radiated EMI Due to PCB Ground Impedance in a 65 W High-Density Active-Clamp Flyback Converter. IEEE Trans. Ind. Electron..

[19] Bhargava A., Pommerenke D., Kam K.W., Centola F., Lam C.W. (2011). DC-DC Buck Converter EMI Reduction Using PCB Layout Modification. IEEE Trans. Electromagn. Compat..

[20] Yen W.-W., Chao P.C.-P. (2022). A ZVS Phase-Shift Full-Bridge Converter with Input Current Steering to Reduce EMI Noise. IEEE Trans. Power Electron..

[21] Safaee A., Jain P.K., Bakhshai A. (2015). An Adaptive ZVS Full-Bridge DC–DC Converter with Reduced Conduction Losses and Frequency Variation Range. IEEE Trans. Power Electron..

[22] Wang Z., Li H., Chu Z., Zhang C., Yang Z., Shao T., Hu Y. (2022). A Review of EMI Research in Modular Multilevel Converter for HVDC Applications. IEEE Trans. Power Electron..

[23] Huang Y., Lin L., Shi X., Yin T., Yan S. (2022). A Trigger Signals Modification Scheme for Common-Mode Conducted EMI Reduction of Modular Multilevel Converter. IEEE J. Emerg. Sel. Top. Power Electron..

[24] Huang H.-H., Chen C.-L., Chen K.-H. (2009). Adaptive window control (AWC) technique for hysteresis DC-DC buck converters with improved light and heavy load performance. IEEE Trans. Power Electron..

[25] Lin Y.-C., Chen C.-J., Chen D., Wang B. (2012). A ripple-based constant on-time control with virtual inductor current and o set cancellation for DC power converters. IEEE Trans. Power Electron..

[26] Pareschi F., Setti G., Rovatti R., Frattini G. (2014). Practical Optimization of EMI Reduction in Spread Spectrum Clock Generators with Application to Switching DC/DC Converters. IEEE Trans. Power Electron..

[27] Pareschi F., Rovatti R., Setti G. (2015). EMI Reduction via Spread Spectrum in DC/DC Converters: State of the Art, Optimization, and Tradeoffs. IEEE Access.

[28] Lin F., Chen D.Y. (1994). Reduction of power supply EMI emission by switching frequency modulation. IEEE Trans. Power Electron.

[29] Gamoudi R., Chariag D.E., Sbita L. (2018). A review of spread spectrum-based PWM techniques—A novel fast digital implementation. IEEE Trans. Power Electron..

[30] Song M., Ahn S., Jung I., Kim Y., Kim C. (2013). Piecewise linear modulation technique for spread spectrum clock generation. IEEE Trans. Large Scale Integr. Syst..

[31] Chang H.H., Hua I.H., Liu S.I. (2003). A spread-spectrum clock generator with triangular modulation. IEEE J. Solid-State Circuits.

[32] Mihali F., Kos D. (2006). Reduced Conductive EMI in Switched-Mode DC–DC Power Converters Without EMI Filters: PWM Versus Randomized PWM. IEEE Trans. Power Electron..

[33] Ho E.N.Y., Mok P.K.T. (2013). Design of PWM Ramp Signal in Voltage-Mode CCM Random Switching Frequency Buck Converter for Conductive EMI Reduction. IEEE Trans. Circuits Syst. I Regul. Pap..

[34] Tao C., Fayed A.A. (2013). PWM Control Architecture with Constant Cycle Frequency Hopping and Phase Chopping for Spur-Free Operation in Buck Regulators. IEEE Trans. Very Large Scale Integr. (VLSI) Syst..

[35] Lee C.F., Mok P.K.T. (2004). A monolithic current-mode CMOS DC-DC converter with on-chip current-sensing technique. IEEE J. Solid-State Circuits.

[36] Rad A.B., Kargaran M., Moosavi S.M.R., Meghdadi M., Medi A. (2024). An Ultra-Low-Noise Buck Converter for Noise-Sensitive Applications. IEEE Trans. Power Electron..

[37] Razavi B. (2018). The Current-Steering DAC [A Circuit for All Seasons]. Solid-State Circuits Mag..

[38] Chen Y., Ma D.B. (2019). EMI-Regulated GaN-Based Switching Power Converter with Markov Continuous Random Spread-Spectrum Modulation and One-Cycle on-Time Rebalancing. IEEE J. Solid-State Circuits.

[39] Texas Instruments (2016). LM5140 Datasheet.

[40] Leoncini M., Bertolini A., Melillo P., Gasparini A., Levantino S., Ghioni M. (2023). Spread-Spectrum Frequency Modulation in a DC/DC Converter with Time-Based Control. IEEE Trans. Power Electron..

[41] Li F., Zhou C., Bao W., Bi C., Zeng P., Du Z. Design and Implementation of a New Hybrid EMI Filter for DC-DC Converter with SiC MOSFETs. Proceedings of the 2023 6th Asia Conference on Energy and Electrical Engineering (ACEEE).

